# Contribution of Trans-Fatty Acid Intake to Coronary Heart Disease Burden in Australia: A Modelling Study

**DOI:** 10.3390/nu9010077

**Published:** 2017-01-18

**Authors:** Jason H. Y. Wu, Miaobing Zheng, Elise Catterall, Shauna Downs, Beth Thomas, Lennert Veerman, Jan J. Barendregt

**Affiliations:** 1 The George Institute for Global Health, Sydney Medical School, University of Sydney, Sydney 2042, Australia; miaobing.zheng@sydney.edu.au (M.Z.); eliseeleven@gmail.com (E.C.); 2 Earth Institute and Institute of Human Nutrition, Columbia University, New York, NY 10025, USA; shauna.downs@sydney.edu.au; 3 National Heart Foundation of Australia, Heart Health & Research, Brisbane 4006, Australia; beth.meertens@heartfoundation.org.au; 4 School of Public Health, the University of Queensland, Brisbane 4006, Australia; l.veerman@sph.uq.edu.au (L.V.); j.barendregt@sph.uq.edu.au (J.J.B.); 5 Epigear International, Sunrise Beach 4567, Australia

**Keywords:** trans-fatty acid, coronary heart disease, Australia, burden, mortality

## Abstract

Trans-fatty acids (TFAs) intake has been consistently associated with a higher risk of coronary heart disease (CHD) mortality. We provided an updated assessment of TFA intake in Australian adults in 2010 and conducted modeling to estimate CHD mortality attributable to TFA intake. Data of the 2011–2012 National Nutrition and Physical Activity Survey was used to assess TFA intake. The CHD burden attributable to TFA was calculated by comparing the current level of TFA intake to a counterfactual setting where consumption was lowered to a theoretical minimum distribution of 0.5% energy. The average TFA intake among adults was 0.59% energy, and overall 10% of adults exceeded the World Health Organization (WHO) recommended limit of 1% energy. Education and income were moderately and inversely associated with TFA intake (*p*-value ≤ 0.001), with one in seven adults in the lowest income and education quintile having >1% energy from TFA. Australia had 487 CHD deaths (95% uncertainty interval, 367–615) due to TFA exposure, equivalent to 1.52% (95% uncertainty limits: 1.15%–1.92%) of all CHD mortality. The relative impact of TFA exposure on CHD mortality in Australia is limited, but, in absolute terms, still substantial. Policies aimed at reducing industrial TFA exposure can reduce socioeconomic inequalities in health and may therefore be desirable.

## 1. Introduction

Trans-fatty acids (TFAs) are unsaturated fatty acids that contain at least one double bond in the trans configuration. TFAs are manufactured via industrial processes (iTFA) including partial hydrogenation and deodorization of vegetable oils, and heating oils at very high temperatures [1]. Low levels of naturally occurring TFAs are also obtained from the meat and milk of ruminant animals (rTFA, e.g., cattle and sheep) [2]. TFAs cause lipid and other metabolic disturbances in experimental studies, and are consistently associated with a higher risk of coronary heart disease (CHD) mortality [3,4]. Around the world, diverse levels of TFA intake have been observed, both due to different dietary habits and varying quantities of iTFA added to processed foods [1]. Limited data from some countries suggest that TFA intakes also vary within populations, and therefore could contribute to health disparities, with higher intakes among population of lower socio-economic status (SES) [5,6]. Given TFAs’ adverse health effects, the World Health Organization (WHO) recommends limiting mean population TFA intake to less than 1% of total energy (%E), and many countries have implemented policies to reduce iTFA in the food supply [7,8]. For example, Denmark passed regulations that stipulate that iTFA cannot exceed 2 g per 100 g of oil or fat in food products (i.e., iTFA should be ≤2% of total fat). However, policies targeting iTFA have shown varying degrees of success, and are often not accompanied by ongoing evaluation of resultant changes in TFA intake and potential impact on CHD mortality [7]. 

In Australia, largely as a result of voluntary reformulation during the 1990s and 2000s encouraged by the government and other health bodies, the use of iTFA is lower than in other countries [9,10]. To continue this improvement in the Australian food supply, independent and government advice sought to phase out the use of iTFA by 2013 [11]. However, the monitoring of TFAs in food products conducted by the Australian food regulatory authority suggests iTFA continues to be present in the Australian food supply [12]. For some Australian processed foods, such as prepared pastry, popcorn, custard baked goods, meat pies and sausage rolls, the percent of TFA as total fat remains high for some products, in some cases exceeding the 2% limit imposed in other countries several folds [12]. To inform public health policy, the aim of this paper is to determine the current Australian intake of TFAs using the latest nationally representative dietary survey data and contemporary information of TFA levels in foods, and to compare the average intake to WHO recommendations. To gain additional insight into the potential relevance and impact of policies targeting TFA, we also examined how TFA intakes vary according to SES, and modeled the proportion of CHD death currently attributable to TFA consumption.

## 2. Materials and Methods

### 2.1. Assessment of TFA Intake in Australia

Data of adults who participated in the 2011–2012 National Nutrition and Physical Activity Survey (NNPAS) were used to assess nationally representative TFA consumption. As part of the 2011–13 Australian Health Survey, the 2011–2012 NNPAS was conducted throughout Australia from May 2011 to June 2012 in approximately 9500 private dwellings (77% of participating dwellings) across Australia to collect dietary intake and physical activity information of the Australian population [13]. A stratified multistage area sampling was used for sample selection to ensure the selected sample was representative of the Australian population. One face-to-face 24 h dietary recall with adults aged 19 years and over was conducted using the five-phase automated multiple-pass method, and used for the analysis of TFA intake. The dietary intake data were coded according to Australian Food, Supplement and Nutrient Database (AUSNUT) food codes. Individual food items were categorized into food classification groups based on the food and measures database developed by Food Standard Australia New Zealand (FASNZ). TFA level for foods in the 2011–2012 NNPAS data was based on the FSANZ 2009 assessment of TFA level in foods [12]. TFA intake (mg/day) and percentage energy from TFA were calculated according to age groups and separately, by sex. Information regarding other sociodemographic variables including education level (bachelor and higher, diploma and certificate, no school qualification, not determined) and income quintiles, were also obtained from the NNPAS survey. Linear regression analyses were conducted to assess the associations between age, gender, education level, and income, and TFA intake (expressed as percent of total energy). Personal weighting factors were applied to the dataset to ensure that the survey estimates conformed to the population estimates by sex, age, area of usual residence and seasonal effects. Statistical analyses were performed using SPSS 20.0 (SPSS Inc., Chicago, IL, USA) with statistical significance set as *p* < 0.05 (two-sided).

### 2.2. CHD Mortality Attributable to TFA

#### 2.2.1. Exposure and Relative Risks

We followed methods previously employed by the Global Burden of Disease (GBD) study to estimate the CHD burden attributable to TFA intake [14]. In essence, potential impact fractions (PIF) were calculated by comparing two scenarios (1) the current level of TFA intake in Australia estimated using the 2011–2012 NNPAS; and (2) a counterfactual (hypothetical) setting where TFA consumption in Australia was lowered to a ‘theoretical minimum distribution’ (TMD). The interpretation of the PIF is the proportion of CHD deaths avoided if the Australian population would always have been exposed to TFA at the level of the theoretical minimum instead of the current observed level. 

To implement this modeling, TFA was treated as a continuous exposure with a lognormal distribution because TFA consumption cannot be negative and the distribution is skewed to the right. We defined the theoretical minimum distribution for TFA with a mean of 0.5%E, with a standard deviation of 0.05, which was the theoretical minimum distribution assumed by the Global Burden of Disease (GBD) nutrition and chronic disease expert group [14,15]. The 0.5%E from TFA was chosen because iTFA is linearly associated with CHD risk and therefore its consumption should be minimized, and if iTFA was eliminated completely from the food supply, then all of the TFA would come from ruminant sources [14,15]. In Australia, 0.5%E from TFA appears to be a reasonable theoretical minimum distribution level—taking the mean of 0.6%E from total TFA currently based on the NNPAS, and the estimates by Food Standard Australia New Zealand (FSANZ) that rTFA contribute up to 75% of total TFA intake in Australia, then elimination of iTFA would leave mean rTFA intake of 0.6% × 75% = ~0.5%E. Globally, this value of theoretical minimum distribution is also consistent with the three lowest TFA intake observed in the GBD study (Barbados, Finland, and Italy, all ≤0.5%E from TFA).

The relation between TFA intake and CHD mortality was modeled as a ‘per unit’ relative risk, which was based on meta-analyses of the observed association between TFA intake and CHD mortality in prospective cohort studies. The relative risks (uncertainty interval) for CHD mortality per 2%E increase in TFA are 1.42 (1.28–1.57) for 25–34 years old, 1.40 (1.27–1.54) for 35–44 years old, 1.33 (1.22–1.45) for 45–54 years old, 1.27 (1.18–1.36) for 55–64 years old, and 1.22 (1.15–1.29) for 65–74 years old and 1.16 (1.11–1.21) for 75 years old and over [14].

We used CHD mortality as assessed by the GBD 2010 study [16]. These numbers are higher than what is reported by the Australian Bureau of Statistics in 2010 because the GBD re-assigns deaths that have been coded to so-called ‘garbage codes’ (such as ‘senility’ or ‘cardiopulmonary arrest’), to what is assumed to be the most likely real cause [16].

#### 2.2.2. Potential Impact Fraction

The potential impact fraction (PIF) calculates the proportional change in disease incidence (or mortality) after a change in risk factor exposure in the population as a whole. It takes risk factor exposure and the relative risk of the disease as its inputs. For a continuous risk factor distribution the equation is [17]:

$$PIF=\frac{\int_{a}^{b}RR\left(x\right)P\left(x\right)dx-\int_{a}^{b}RR\left(x\right)P^{*}\left(x\right)dx}{\int_{a}^{b}RR\left(x\right)P\left(x\right)dx}$$

where *x* is the risk factor level, *P*(*x*) is the original risk factor distribution, P*(*x*) is the risk factor distribution after the change, *RR*(*x*) is the relative risk as a function of risk factor exposure level, dx denotes that the integration is done over x, and a and b are the integration boundaries. For the present study, we calculated PIFs by age group and sex with the observed distributions of TFA exposure and the TMD. The *RR*(*x*) function is implemented using the aforementioned “per unit” relative risks, which implies that the risk increases exponentially with increasing exposure.

#### 2.2.3. Calculation Methods and Uncertainty

Calculations were conducted in Microsoft Excel (Microsoft, Redmond, WA, USA), with the help of two add-in programs. The EpigearXL add-in provides functions to calculate the integrals of the multiplication of a lognormal distribution with a “per unit” relative risk function. The Ersatz add-in allows doing Monte Carlo simulation in Excel. Both add-in programs are available from Epigear website [18]. Two of the inputs, the relative risks and the exposure have sampling uncertainty. We assume, by virtue of the central limit theorem, that the mean exposure has a normal distribution with the standard error of the mean as its standard deviation. For the relative risks we used Ersatz’s ErRelativeRisk function [19]. The 2010 CHD mortality numbers have uncertainty as well [16], but since these are population numbers the uncertainty is not sampling-related (it pertains to coding practices and such) and it is therefore not included in the uncertainty analysis. We did a Monte Carlo simulation with 2000 iterations to quantify the effect of the sampling uncertainty on the attributable mortality.

## 3. Results

### 3.1. TFA Intake

The average TFA intake among adults ≥19 years of age was 1.39 ± 1.14 g/day (mean ± standard deviation, which equates to 0.59%E ± 0.38%E. The 95th percentile was ~3.6 g/day. Table 1 illustrates TFA intake by age- and gender-specific groups. TFA intake was generally similar between men and women across the age groups. The 90th percentile for TFA intake was ~1%E in both men and women, i.e., the usual intake of TFA is >1%E for about 10% of Australian adults. 

TFA intake differed according to the level of education and income (Table 2). Participants with a higher education and more income had significantly lower levels of TFA intake. For example, the average TFA intake was ~10% lower among participants with a Bachelor or higher degree compared to those with no school qualification. In a multivariate model adjusting for age, gender, education and income, both education and income were significantly associated with TFA intake (*p*-value≤ 0.001 for both). The percentages of participants who exceeded the 1%E in the lowest level of income and lowest level of education were 14.2% (253 out of 1776) and 14.1% (515 out of 3636) compared to 10% overall for Australian adults.

### 3.2. CHD Mortality Attributable to TFA

Table 3 shows the results of the TFA-attributable CHD mortality for the base year of 2010. The attributable deaths were overwhelmingly in the 75+ age group, but more so for women (82% of attributable death in the 75+ age group) than for men (62%). Overall, Australia had 487 CHD deaths (95% CI, 367–615 CHD deaths) due to TFA exposure in 2010. This is about 1.52% (95% uncertainty limits: 1.15%–1.92%) of all CHD mortality.

## 4. Discussion

These findings demonstrate that about one in 10 Australians continue to exceed the WHO guidelines for limiting TFA consumption to less than 1%E. There was a moderate association between SES and TFA consumption, and those with less income and education consumed more TFAs. As a result, about one in seven Australians from the lowest income and education quintiles exceeded the 1%E. At the 2010 population intake level, we estimated that approximately 1.5% of CHD deaths were attributable to TFA.

Systematic review and meta-analyses consistently support the adverse metabolic effects of iTFA and the association with a higher CHD risk [20,21]. In Australia, voluntary reformulation by the food industry has led to notable reductions in iTFA in key product categories such as margarines through the 1990s and 2000s [22], and the use of iTFA appeared to be lower than in other countries [9,10]. However, there has been ongoing concern and debate by the Australian government and other health advocates regarding the adequacy of current policies, and if additional regulations are needed to monitor and phase out iTFA in the food supply [11]. The estimated mean TFA intake in Australia (0.6%E) is indeed relatively low in comparison to other countries such as Brazil [23], Canada [24], Iran [25], the Netherlands [26], the UK [27,28] and the US [29] (0.8%E–4.2%E, Supplementary Materials Table S1). However, interpreting our findings in the historical context in Australia suggests there has been little meaningful change in mean TFA intake in Australia over the past decade. Serial surveys conducted by the Australian government have found largely comparable TFA values across major dietary sources between 2005 and 2013 [12]. These surveys indicate iTFA continues to be present in commonly consumed products, and there has been no consistent trend in the reduction of surveyed product categories over the previous decade [12]. Our findings highlight that while the proportion of CHD burden attributable to TFA intake is relatively small, in absolute terms, this still equates to almost 500 deaths in 2010 because CHD is a major cause of death in Australia; further, that CHD burden is more likely to be carried by those with less income and education. These modeling results add to the evidence base, and should be taken into consideration by policy-makers in future deliberations around regulatory actions aimed at reducing TFAs. A detailed cost-effectiveness analysis was outside the scope of the current investigation, but it should be conducted in future studies to aid decision-makers in Australia who need to consider public health benefits as well as cost to the food industry related to the implementation of new policies to reduce iTFA. Policy-making should also take into account the evidence available in other countries such as Denmark, where mandated limits have been successfully implemented and have virtually eliminated iTFA from the food supply. 

It should be noted that our modeling of TFA intake uses NNPAS data which uses the ‘average’ TFA level for a product category. While this is accepted methodology for estimating the average TFA intake at the population level, it will not identify sub-groups who are exposed to high levels of TFA due to their preference or regular consumption of high-TFA products. The extent to which a high TFA intake is possible in such subgroups in Australia is demonstrated in Supplementary Materials Table S2, using a hypothetical ‘high-TFA menu’ [2]. The continued existence of specific products with high TFA levels suggests that daily consumption of ~7 g/day (~3%E) of TFA is possible. High levels of TFA intake in subgroups of susceptible populations are most likely addressed via bans or restrictions of the level of iTFA allowed in foods, as has been observed in countries such as Denmark [2].

Prior studies have established that poor dietary quality and its associated diseases are strongly socio-economically patterned, and there is a need for food policy interventions that improve inequalities in dietary quality [30]. Adults from lower SES backgrounds tend to consume more processed and discretionary products, which likely accounts for the higher intake of TFA in this group in Australia [31]; this is also consistent with observations in the UK and the US [32,33]. A systematic review of food policies targeting TFA globally has identified policies including mandatory labeling, voluntary reformulation, and bans [7], and modeling studies in other countries have suggested that compared to mandatory labeling, limits or bans on iTFA have the greatest potential to reduce the CHD risk for the most disadvantaged groups and thereby reduce health inequalities [5,32]. Our findings highlight the need for additional investigations to model the potential impact on health inequalities of different policy approaches to further reduce iTFA in Australia.

A key strength of the current paper is that we utilized the most up-to-date, individual-level, nationally representative NNPAS dietary survey data from 2011–2012, which also took into account the level of TFA in different foods using the relatively recent 2009 FSANZ TFA in foods survey. We also adapted well-validated methodology to estimate the CHD burden attributable to TFA consumption. Our study represents an updated analysis of a prior GBD analysis, which found a much higher percentage of CHD mortality attributable to TFA: 5.9% (95% CI, 5.5%–6.4%) [14]. The difference seems entirely due to a much higher estimate of TFA exposure (1.3% vs. 0.6% of TFA intake in 2010). The large difference in the estimated mean TFA consumption in 2010 is likely due to major methodologic differences in dietary assessment methods. In contrast to our approach, the GBD study estimated TFA intake in Australia using statistical modeling, which relied on inputs that included the 1995 Australian Dietary survey, country- and region-level covariates such as lagged distributed income, and food disappearance data [14,15]. Authors of the GBD paper noted that a limitation of their study was that TFA consumption data was relatively limited in most nations compared with other major dietary factors [14,15]. While the NNPAS survey has its own methodologic challenges, it is likely to more accurately quantify the current mean TFA consumption in Australia.

A number of limitations should be noted. The estimate in this report calculates attributable deaths using PIFs, and due to its underlying assumptions, the methodology does not take into account competing risk factors and overestimates the proportion of death attributable to a single risk factor. As a consequence, our attributable deaths will be overestimated, although this effect is rather small for small risk factors such as TFA. A second reason why PIFs cause overestimation is that it is a static calculation, i.e., it assumes that the counterfactual population has always been exposed to the theoretical minimum level of TFA. In a real population, a one-off change from the current level to the theoretical minimum would imply time lags in the decrease in the heart incidence disease risk, and longer ones (because of the existing pool of prevalent cases) in CHD mortality. In comparative risk assessment terms, this would be the avoidable burden, as opposed to the attributable burden that we estimated in this paper. It should be noted that attributable deaths only captures one dimension of the health impact of TFA consumption, but does not inform other important public health parameters including age at death, life years lost, morbidity, and health-adjusted years of life lost, which are outside the scope of the current study but should be the focus of future analyses. Engagement with policy-makers to assess their views and acceptance of strengthened policies to further reduce iTFA in the food supply should also be a focus for future studies. The disease burden of other forms of cardiovascular disease (e.g., stroke) attributable to TFA was not modeled in this study, but could be included in future analyses if evidence for causal relationships becomes stronger. In addition to random measurement errors, under-reporting was likely present in the NNPAS [13]. Furthermore, people may selectively under-report intake of discretionary foods (which are common sources of iTFA) for social desirability reasons [34], which could potentially cause bias towards a lower estimated TFA intake. 

## 5. Conclusions

In conclusion, the relative impact of TFA exposure on CHD mortality in Australia is limited, but in absolute terms still substantial. Policy-making to reduce iTFA exposure may therefore be desirable, especially as, currently, TFA levels continue to be high in some food products in Australia. Comprehensive, high-quality, and periodic surveys need to continue to monitor levels of TFA in the food supply. Such data will be required to inform policy-makers regarding the efficacy of policies to reduce iTFA.

## Figures and Tables

**Table 1:** Trans-fatty acid intake in Australia based on the 2011–2012 National Nutrition Physical Activity Survey *.

Age (Years)	19–24		25–34		35–44		45–54		55–64		65–74		75+	
Gender	Men	Women	Men	Women	Men	Women	Men	Women	Men	Women	Men	Women	Men	Women
*n*	560	526	878	879	873	882	830	855	705	725	459	478	307	384
	TFA intake as percent of total energy													
Mean ± standard deviation	0.59 ± 0.35	0.57 ± 0.39	0.56 ± 0.36	0.59 ± 0.39	0.58 ± 0.33	0.55 ± 0.33	0.56 ± 0.34	0.56 ± 0.37	0.61 ± 0.41	0.56 ± 0.40	0.59 ± 0.38	0.58 ± 0.39	0.65 ± 0.41	0.64 ± 0.42
90th percentile	0.97	1.02	1.04	1.09	1.02	1.04	1.00	0.97	1.21	1.12	1.05	1.00	1.21	1.11
95th percentile	1.18	1.46	1.29	1.29	1.19	1.24	1.21	1.20	1.45	1.30	1.23	1.38	1.42	1.36

* TFA intake was expressed as percent of total energy intake (%E).

**Table 2:** Trans-fatty acid intake in Australia according to education and income levels based on the 2011–2012 National Nutrition Physical Activity Survey *.

Education Level	No school Qualification (*n* = 3636)	Diploma and Certificate (*n* = 3177)	Bachelor and Higher (*n* = 2385)			*p*-Trend ^†^
Mean ± standard deviation (SD)	0.60 ± 0.39	0.59 ± 0.38	0.54 ± 0.32			<0.0001
90th percentile	1.12	1.05	0.95			
95th percentile	1.30	1.32	1.15			
Income quintiles (Q1 lowest, Q5 highest)	Q1 (*n* = 1776)	Q2 (*n* = 1531)	Q3 (*n* = 1530)	Q4 (*n* = 1812)	Q5 (*n* = 1776)	*p*-trend ^†^
Mean ± SD	0.62 ± 0.42	0.60 ± 0.39	0.57 ± 0.36	0.56 ± 0.34	0.56 ± 0.34	0.001
90th percentile	1.16	1.08	1.06	1.00	0.99	
95th percentile	1.45	1.32	1.29	1.21	1.21	

* *n* = 143 and 916 participants did not have data on education and income, respectively, and were excluded from these analyses; ^†^ Based on multivariate regression adjusting for age, gender, education, and income. Education and income groups were coded sequentially as 1, 2, 3, etc., and analyzed as continuous variables to obtain the *p*-values for the trend.

**Table 3:** Coronary heart disease mortality attributable to TFA in 2010, by sex and age group and in total, with 95% uncertainty intervals.

	Males	95% UI		Females	95% UI		Total	95% UI	
Age (Years)	Mean	LUI	UUI	Mean	LUI	UUI	Mean	LUI	UUI
25–34	1	1	1	0	0	1	1	1	2
35–44	5	4	7	1	1	2	7	5	9
45–54	14	10	19	3	2	4	18	12	23
55–64	34	23	44	8	5	11	42	28	54
65–74	45	31	59	20	14	26	65	45	85
75+	161	108	221	193	128	263	355	239	480
Total	260	202	326	226	162	297	487	367	615

UI: uncertainty interval, LUI: lower uncertainty interval, UUI: upper uncertainty interval.

## Supplementary Materials

The following are available online at http://www.mdpi.com/2072-6643/9/1/077/s1, Table S1. TFA intake in Australia vs. other countries; Table S2. Total amount of TFA obtained from a hypothetical ‘high-TFA menu’.
