# Supplementary material for: Contribution of Trans-Fatty Acid Intake to Coronary Heart Disease Burden in Australia: A Modelling Study
Source: Nutrients. 2017 Jan 18;9(1):77. doi: 10.3390/nu9010077 (PMC5295121; doi:10.3390/nu9010077)
Supplement: Supplementary file 1 [file nutrients-09-00077-s001.docx]

Supplementary Material: Contribution of Trans-Fatty Acid Intake to Coronary Heart Disease Burden in Australia: A Modelling Study

Jason H.Y. Wu, Miaobing Zheng, Elise Catterall, Shauna Downs, Beth Thomas, Lennert Veerman and Jan J. Barendregt

**Table S1.** Trans-fatty acid (TFA) intake in Australia vs. other countries *.

| **Countries and Year of Dietary Survey** | **TFA Intake as % of Energy** | | **Survey Methods and Population** |
| --- | --- | --- | --- |
|  | mean | SD |  |
| Australia, 2013 (Current analysis) | 0.6 | 0.4 | One-day 24-h diet recall, nationally representative population survey |
| Brazil, 2008–2009 [1] | 1.4 | - | Two-day food diary, nationally representative population survey |
| Canada, 2004 [2] | 1.4 | - | 24-h diet recall, nationally representative population survey |
| Iran, 2001–2003 [3] | 4.2 | - | Three consecutive 24-h dietary recalls, nationally representative population survey |
| Netherlands, 2003 [4] | 0.8 | - | 24-h diet recall, sample aged 19–30 |
| UK, 2008–2011 [5,6] | 0.8 | 0.4 | Four-day food diary, nationally representative population survey |
| US, 2007–2009, Men [7] | 1.9 | - | One-day 24-h diet recall, cross-sectional survey of adults in Minneapolis |
| US, 2007–2009, Women [7] | 1.7 | - | One-day 24-h diet recall, cross-sectional survey of adults in Minneapolis |

* A search was conducted via Pubmed to identify published literature since the year 2000 that reported TFA intake in adult populations to enable comparison against the current analysis. SD: standard deviation.

**Table S2.** Total amount of trans-fatty acid (TFA) obtained from a hypothetical ‘high TFA menu *.

| **Product** | **Weight of Product Consumed Per Day (g)** | **TFA (g/100 g Product) *** | **TFA Consumed (g)** |
| --- | --- | --- | --- |
| Meat Pie | 175 | 0.96 | 1.68 |
| Custard baked goods | 100 | 0.52 | 0.52 |
| Popcorn | 100 | 4.83 | 4.83 |
| Total |  |  | 7.03 |

* Based on Food Standard Australia New Zealand (FSANZ) 2013 analyses of TFA level in 500 food samples. Products with the highest TFA level in each of the product category were selected for this hypothetical menu.

References

1. Pereira, R.A.; Duffey, K.J.; Sichieri, R.; Popkin, B.M. Sources of excessive saturated fat, trans fat and sugar consumption in Brazil: An analysis of the first Brazilian nationwide individual dietary survey. *Public Health Nutr.* 2014, *17*, 113–121.
2. Ratnayake, W.M.; L’Abbe, M.R.; Farnworth, S.; Dumais, L.; Gagnon, C.; Lampi, B.; Casey, V.; Mohottalage, D.; Rondeau, I.; Underhill, L.; et al. Trans fatty acids: Current contents in Canadian foods and estimated intake levels for the Canadian population. *J. AOAC Int.* 2009, *92*, 1258–1276.
3. Mozaffarian, D.; Abdollahi, M.; Campos, H.; Houshiarrad, A.; Willett, W.C. Consumption of trans fats and estimated effects on coronary heart disease in Iran. *Eur. J. Clin. Nutr.* 2007, *61*, 1004–1010.
4. Temme, E.H.; Millenaar, I.L.; Van Donkersgoed, G.; Westenbrink, S. Impact of fatty acid food reformulations on intake of Dutch young adults. *Acta Cardiol.* 2011, *66*, 721–728.
5. Pot, G.K.; Prynne, C.J.; Roberts, C.; Olson, A.; Nicholson, S.K.; Whitton, C.; Teucher, B.; Bates, B.; Henderson, H.; Pigott, S.; et al. National diet and nutrition survey: Fat and fatty acid intake from the first year of the rolling programme and comparison with previous surveys. *Br. J. Nutr.* 2012, *107*, 405–415.
6. Roe, M.; Pinchen, H.; Church, S.; Elahi, S.; Walker, M.; Farron-Wilson, M.; Buttriss, J.; Finglas, P. Trans fatty acids in a range of UK processed foods. *Food Chem.* 2013, *140*, 427–431.
7. Honors, M.A.; Harnack, L.J.; Zhou, X.; Steffen, L.M. Trends in fatty acid intake of adults in the Minneapolis-St Paul, MN metropolitan area, 1980–1982 through 2007–2009. *J. Am. Heart Assoc.* 2014, *3*, e001023.
